# TyG index is positively associated with risk of CHD and coronary atherosclerosis severity among NAFLD patients

**DOI:** 10.1186/s12933-022-01548-y

**Published:** 2022-07-01

**Authors:** Jianqi Zhao, Hongxuan Fan, Ting Wang, Bing Yu, Shaobin Mao, Xun Wang, Wenjing Zhang, Leigang Wang, Yao Zhang, Zhaoyu Ren, Bin Liang

**Affiliations:** 1 Department of Cardiology, The Second Hospital of Shanxi Medical University, 382 Wuyi Road, Taiyuan, 030001 Shanxi China; 2 Department of Neurology, The Second Hospital of Shanxi Medical University, 382 Wuyi Road, Taiyuan, 030001 Shanxi China

**Keywords:** Triglyceride-glucose index, Insulin resistance, Non-alcoholic fatty liver diseases, Coronary heart disease

## Abstract

**Background:**

Insulin resistance (IR), endothelial dysfunction, inflammation, glucose and lipid metabolism disorders, and thrombosis are believed involved in coronary heart disease (CHD) and non-alcoholic fatty liver disease (NAFLD). Triglyceride-glucose (TyG) index, a new IR indicator, is correlated with NAFLD occurrence and severity, but its relationship with CHD risk remains unclear. This study investigated the correlation between TyG index and CHD risk among NAFLD patients.

**Methods:**

This cross-sectional study included 424 patients with NAFLD and chest pain in the Department of Cardiology, The Second Hospital of Shanxi Medical University, from January 2021 to December 2021. The TyG index was calculated and coronary angiography performed. All individuals were divided into NAFLD + CHD and NAFLD groups and then by TyG index level. The t-test, Mann–Whitney U-test, or one-way analysis of variance compared differences in continuous variables, while the chi-square test or Fisher’s exact test compared differences in categorical variables. Logistic regression analysis determined the independent protective or hazardous factors of NAFLD with CHD. The receiver operating characteristic curve evaluated the ability of different TyG index rule-in thresholds to predict CHD. The relationship between Gensini score and TyG index was evaluated using linear correlation and multiple linear regression.

**Results:**

CHD was detected in 255 of 424 patients. Compared to NAFLD group, multivariate logistic regression showed that TyG index was a risk factor for CHD among NAFLD patients after adjustment for age, sex, hypertension, and diabetes mellitus with the highest odds ratio (OR, 2.519; 95% CI, 1.559–4.069; P < 0.001). TG, low-density lipoprotein cholesterol, FBG and TYG–body mass index were also risk factors for CHD among NAFLD patients. High-density lipoprotein cholesterol level was a protective factor for CHD events in patients with NAFLD. In an in-depth analysis, multivariate logistic regression analysis showed that each 1-unit increase in TyG index was associated with a 2.06-fold increased risk of CHD (OR, 2.06; 95% CI, 1.16–3.65; P = 0.013). The multifactor linear regression analysis showed each 0.1-unit increase in TyG in the NAFLD-CHD group was associated with a 2.44 increase in Gensini score (β = 2.44; 95% CI, 0.97–3.91; P = 0.002).

**Conclusions:**

The TyG index was positively correlated with CHD risk in NAFLD patients and reflected coronary atherosclerosis severity.

## Background

Coronary heart disease (CHD) is the most common cardiovascular disease (CVD) and a major cause of chronic disease–related deaths worldwide. Atherosclerotic plaque formation and development is the most important pathophysiological process in CHD [1]. Possible mechanisms include endothelial cell injury, inflammation, oxidative stress, glucose and lipid metabolism disorders, and thrombosis [2]. Recent studies reported that insulin resistance (IR) is involved in coronary plaque formation and remodeling independent of traditional risk factors such as age, smoking, and hypertension (HTN) [3].

Non-alcoholic fatty liver disease (NAFLD), an important global public health problem, is a manifestation of metabolic syndrome in the liver and a major risk factor for CVD [4, 5]. An increasing number of studies have shown that NAFLD is associated with higher mortality rates from cardiovascular events, and IR is believed to play an important role in cardiovascular events in patients with NAFLD; however, the role of other atherosclerotic mechanisms should not be ignored [6–8].

The triglyceride-glucose (TyG) index is an emerging index reflecting IR because of its consistency with the high insulin-glucose clamp test, the current gold standard for IR diagnosis [9, 10]. Recent studies reported that the TyG index is also associated with inflammation, endothelial dysfunction, glucolipid metabolism disorders, thrombosis, and other atherosclerotic factors [11–13]. Previous studies confirmed that the TyG index can predict NAFLD occurrence and severity [14, 15]. However, the correlation between the TyG index and the risk of CHD in patients with NAFLD remains unclear. Therefore, this study aimed to determine the correlation between the TyG index and the occurrence of CHD and coronary artery disease severity in NAFLD patients.

## Methods

### Subjects and study design

This cross-sectional study included 424 patients with NAFLD who underwent coronary angiography at the Second Affiliated Hospital of Shanxi Medical University for chest pain between January and December 2021. All participants were divided into NAFLD + CHD and NAFLD groups based on the coronary angiography results.

The Judkin method was used to record the coronary angiography results, while the degree of coronary artery stenosis was quantified as Gensini score [16] as lumen diameter stenosis ≥ 50% in any of the main coronary arteries or other important branches [17].

NAFLD was diagnosed according to the recommendations of the Asia–Pacific Working Party recommendations [18]: the presence of fatty liver, exclusion of excessive alcohol consumption (> 140 g/week for men and > 70 g/week for women), history of viral hepatitis, and utilization of hepatotoxic drugs. The liver was assessed for hepatic steatosis by ultrasound by a professional operator using the standard method of enhanced liver echo versus the renal cortex.

Subjects with a previous history of using statins or triglyceride-lowering drugs, CHD, percutaneous coronary intervention or coronary artery bypass grafting, or other heart diseases such as rheumatic heart disease, valvular heart disease, cardiac syndrome X, severe congenital heart disease, cardiomyopathy, severe heart failure, etc. were excluded. We also excluded subjects with malignant tumors, autoimmune diseases, acute or chronic infectious diseases, or serious cerebrovascular accidents.

This study was approved by the Ethics Committee of the Second Hospital of Shanxi Medical University. All participants provided written informed consent.

### Data collection and measurements

The patients’ general clinical data such as sex, age, height, weight, systolic blood pressure, diastolic blood pressure (DBP), HTN, diabetes mellitus (DM), and smoking history were collected through electronic medical record review.

Fasting cubital vein blood (3 mL) was collected the morning of the day after admission and placed in an anticoagulant tube for examination. Alanine aminotransferase (ALT), aspartate aminotransferase (AST), serum creatinine (Scr), total cholesterol (TC), triglyceride (TG), high-density lipoprotein cholesterol (HDL-C), low-density lipoprotein cholesterol (LDL-C), fasting blood glucose (FBG), and other biochemical indicators were detected using enzyme-linked immunosorbent assay. We then calculated body mass index (BMI), TyG index, and TyG-BMI index. BMI was defined as weight (in kilograms)/height squared (in meters), TyG index was calculated using the formula Ln [TG (mg/dL) × FBG (mg/dL)/2] [19], and TyG-BMI was computed by TyG index × BMI.

### Statistical analysis

All data were analyzed using IBM SPSS Statistics version 23.0 (Chicago, IL, USA), GraphPad Prism 8.0 (San Diego, California, USA), and Free Statistics version 1.4 (Beijing, China). The normality of the continuous variable distribution was evaluated using the Shapiro–Wilk test. Normally distributed data are expressed as mean and standard deviation, while non-normally distributed data are expressed as median with interquartile range. Categorical variables are described as frequency and percentage (%). Normally distributed values were compared between NAFLD groups with and without CHD using unpaired Student’s t-tests. Non-normally distributed values were compared using Mann–Whitney tests between the NAFLD groups with and without CHD.

Potential predictors of CHD were initially investigated using univariate logistic regression analysis, followed by multivariate analysis to identify independent predictors and their power. The baseline characteristics of the TyG groups were tested with the t-test or one-way analysis of variance for continuous variables and the chi-square or Fisher’s exact test for categorical variables. Uni- and multivariate logistic regression analyses were performed to determine the effect of different TyG levels on the risk of CHD. The ability of different TyG index rule-in thresholds to predict CHD was evaluated by receiver operating characteristic (ROC) curve analysis and area under the curve (AUC) values. Linear correlation and multiple linear regression analyses were conducted to evaluate the relationship between Gensini scores and the TyG index.

## Results

### Clinical and biochemical characteristics in NAFLD and NAFLD-CHD groups

Data were generated from 424 patients with NAFLD (266 men and 158 women), including 255 participants with CHD and 169 participants without CHD. The mean age of individuals were 58.5 ± 10.8 and 55.0 ± 9.9 years in the NAFLD with and without CHD groups. Table 1 shows the demographic and clinical characteristics of the two groups. The two groups of participants did not differ significantly in terms of BMI, systolic blood pressure, DBP, and TC. Meanwhile, the NAFLD with CHD group had a higher proportion of male participants, smokers, those with HTN and DM; higher mean participant age; higher mean ALT, AST, Scr, TG, FBG, TyG, and TyG-BMI levels; and lower HDL-C levels (all P < 0.05).

**Table1:** Demographic and clinical characteristics of participants by the presence of CHD

	CHD + NAFLD (n = 255)	NAFLD (n = 169)	*t/χ*^*2*^*/Z*	*P*
Gender (male)	178 (69.8)	88 (52.1)	13.671	< 0.001
Age (years)	58.5 ± 10.8	55.0 ± 9.9	3.381	0.001
BMI (kg/m^2^	26.3 ± 3.0	26.2 ± 2.9	0.507	0.612
Smoke [(n%)]	135 (52.9)	52 (30.8)	20.268	< 0.001
HT [(n%)]	173 (67.8)	80 (47.3)	17.760	< 0.001
DM [(n%)]	100 (39.2)	24 (14.2)	30.735	< 0.001
SBP (mmHg)	133.6 ± 19.4	131.9 ± 17.1	0.904	0.367
DBP (mmHg)	81.4 ± 13.0	80.4 ± 11.9	0.779	0.436
ALT (U/L)	26.3 (18.6–35.4)	22.3 (16.2–31.9)	− 2.425	0.015
AST (U/L)	23.8 (19.1–29.7)	21.7 (18.3–25.5)	− 2.585	0.010
Scr (mmol/L)	67.3 ± 13.7	64.5 ± 11.6	2.177	0.030
TC (mmol/L)	4.47 ± 1.11	4.37 ± 0.98	0.914	0.361
TG (mmol/L)	1.81 (1.33–2.60)	1.62 (1.20–2.21)	− 3.040	0.002
HDL-C (mmol/L)	1.08 ± 0.25	1.18 ± 0.27	− 3.987	< 0.001
LDL-C (mmol/L)	2.53 ± 0.72	2.29 ± 0.70	3.521	< 0.001
FGB (mmol/L)	5.86 (4.99–7.37)	5.29 (4.91–5.83)	− 4.828	< 0.001
TyG	9.10 (8.76–9.53)	8.86 (8.53–9.17)	− 5.263	< 0.001
TyG-BMI	239.6 (219.0–261.7)	228.3 (215.6–246.8)	− 3.169	0.002

Data are shown as means ± SD or median (quartile). Normal values were compared using paired and unpaired Student’s t-tests, and non-normally distributed values were compared using Wilcoxon rank-sum or Mann–Whitney tests. Chi-square test was used to classification variables

*BMI* body mass index, *HT* hypertension, *DM* diabetes mellitus, *SBP* systolic blood pressure, *DBP* diastolic blood pressure, *ALT* alanine aminotransferase, *AST* aspartate aminotransferase, *Scr* serum creatinine, *TC* total cholesterol, *TG* triglycerides, *HDL-C* high-density lipoprotein cholesterol, *LDL-C* low-density lipoprotein cholesterol, *FGB* fasting plasma glucose, *TyG* triglyceride-glucose index, *TyG-BMI* TyG index with body mass index

### Univariate and multivariate analyses of factors associated with CHD in NAFLD

**Fig. 1:**
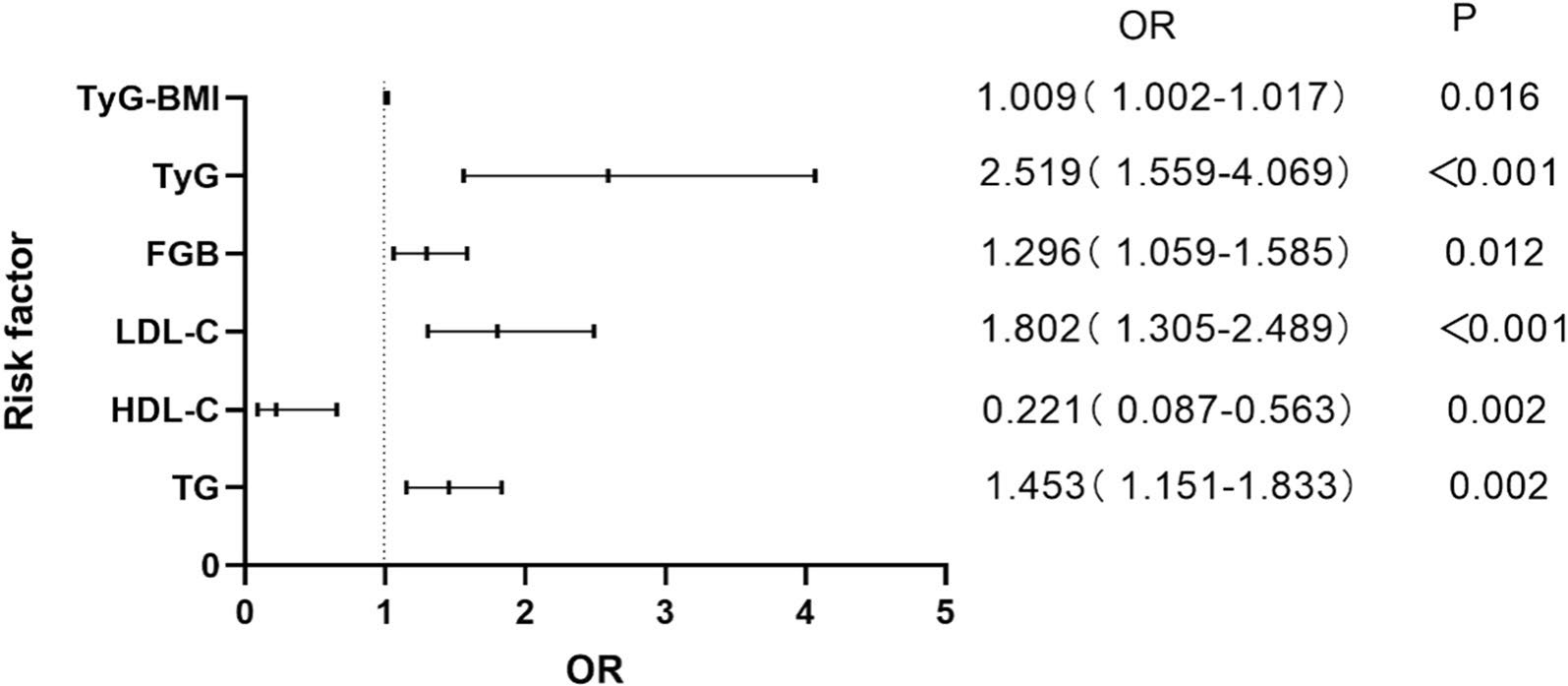
Forest plots of independent factors associated with CHD in NAFLD

The univariate logistic regression analysis showed that Scr, TG, HDL-C, LDL-C, FBG, TyG, and TyG-BMI were correlated with the occurrence of CHD in NAFLD patients. The multivariate logistic regression analysis showed that Scr was not a risk factor for CHD in NAFLD patients, whereas TG (OR, 1.453; 95% CI, 1.151–1.833; P = 0.002), LDL-C (OR, 1.802; 95% CI, 1.305–2.489; P < 0.001), FBG (OR, 1.296; 95% CI, 1.059–1.585; P = 0.012), TyG (OR, 2.519; 95% CI, 1.559–4.069; P < 0.001) and TyG-BMI (OR, 1.009; 95% CI, 1.002–1.017; P = 0.016) were still risk factors for CHD in NAFLD patients. HDL-C (OR, 0.221; 95% CI, 0.087–0.563; P = 0.002) was a protective factor for CHD (Table 2; Fig. 1).

**Table 2:** Univariate and multivariate analyses of factors associated with CHD in NAFLD

	Non-adjusted		Model I		Model II	
	OR (95%CI)	*P*	OR (95%CI)	*P*	OR (95%CI)	*P*
BMI (kg/m^2^)	1.017 (0.952–1.806)	0.611				
SBP (mmHg)	1.005 (0.994–1.016)	0.366				
DBP (mmHg)	1.006 (0.991–1.022)	0.435				
ALT (U/L)	1.006 (0.992–1.020)	0.395				
AST (U/L)	1.017 (0.994–1.042)	0.147				
Scr (mmol/L)	1.017 (1.002–1.033)	0.031	0.996 (0.977–1.015)	0.681		
TC (mmol/L)	1.090 (0.906–1.311)	0.360				
TG (mmol/L)	1.452 (1.179–1.787)	< 0.001	1.523 (1.221–1.901)	< 0.001	1.453 (1.151–1.833)	0.002
HDL-C (mmol/L)	0.217 (0.099–0.474)	< 0.001	0.180 (0.075–0.431)	< 0.001	0.221 (0.087–0.563)	0.002
LDL-C (mmol/L)	1.657 (1.241–2.212)	0.001	1.740 (1.284–2.360)	< 0.001	1.802 (1.305–2.489)	< 0.001
FGB (mmol/L)	1.467 (1.257–1.714)	< 0.001	1.447 (1.228–1.705)	< 0.001	1.296 (1.059–1.585)	0.012
TyG	3.197 (2.097–4.874)	< 0.001	3.218 (2.075–4.992)	< 0.001	2.519 (1.559–4.069)	< 0.001
TyG-BMI	1.011 (1.004–1.018)	0.001	1.013 (1.006–1.021)	< 0.001	1.009 (1.002–1.017)	0.016

None, non-adjusted model. Model I was adjusted for age and sex. Model II was adjusted for age, sex, hypertension, diabetes mellitus and smoking history

### Clinical and biochemical characteristics by TyG index

To understand the relationship between different TyG levels and CHD in NAFLD patients, we divided the patients into tertiles according to TyG index (tertile 1: n = 141, TyG index ≤ 8.77; tertile 2: n = 141, 8.78 ≤ TyG index ≤ 9.22; tertile 3: n = 142, TyG index ≥ 9.22). The average TyG index in the three groups was 8.5 ± 0.2, 9.0 ± 0.1, and 9.7 ± 0.5. Statistically significant differences were noted among the three groups in rates of CHD, DM, ALT, AST, TC, TG, HDL-C, LDL-C, FBG, and TYG-BMI (all P < 0.05) (Table 3).

**Table 3:** Demographic and clinical characteristics of participants by TyG index

	Tertile 1 (n = 141)	Tertile 2 (n = 141)	Tertile 3 (n = 142)	*t/χ*^*2*^*/F*	*P*
Gender (male)	80 (56.7)	89 (63.1)	97 (68.3)	4.066	0.131
Age (years)	57.4 ± 10.3	56.7 ± 10.5	57.1 ± 11.0	0.151	0.86
BMI (kg/m^2^)	26.0 ± 2.6	26.7 ± 3.2	26.2 ± 3.1	0.614	0.142
Smoke [(n%)]	53 (37.6)	64 (45.4)	70 (49.3)	4.075	0.13
HT [(n%)]	77 (54.6)	82 (58.2)	94 (66.2)	4.148	0.126
DM [(n%)]	18 (12.8)	34 (24.1)	72 (50.7)	51.9	< 0.001
SBP (mmHg)	131.8 ± 16.4	134.1 ± 17.0	132.9 ± 21.7	0.55	0.577
DBP (mmHg)	80.3 ± 10.5	81.2 ± 13.4	81.4 ± 13.7	0.276	0.759
ALT (U/L)	22.2 (16.6, 30.8)	24.9 (17.4, 33.6)	29.4 (19.1, 36.3)	9.425	0.009
AST (U/L)	22.3 (18.4, 27.1)	22.3 (18.4, 26.5)	23.9 (20.3, 30.6)	6.496	0.039
SCr (mmol/L)	67.0 (57.0, 75.0)	66.0 (56.0, 75.0)	66.0 (56.2, 76.0)	0.244	0.885
TC (mmol/L)	4.1 ± 1.0	4.3 ± 1.0	4.9 ± 1.1	19.423	< 0.001
TG (mmol/L)	1.2 (1.1, 1.4)	1.8 (1.5, 2.1)	2.7 (2.2, 3.8)	264.246	< 0.001
HDL-C (mmol/L)	1.2 ± 0.3	1.1 ± 0.2	1.0 ± 0.3	20.456	< 0.001
LDL-C (mmol/L)	2.2 ± 0.7	2.4 ± 0.7	2.7 ± 0.7	14.511	< 0.001
FGB (mmol/L)	5.2 (4.8, 5.6)	5.6 (4.9, 6.4)	6.5 (5.4, 8.3)	77.996	< 0.001
TyG	8.5 ± 0.2	9.0 ± 0.1	9.7 ± 0.5	464.341	< 0.001
TyG-BMI	220.9 ± 22.7	239.7 ± 29.7	254.5 ± 32.2	49.574	< 0.001
CHD [(n%)]	68 (48.2)	82 (58.2)	105 (60.1)	19.866	< 0.001

Data are presented as mean ± standard deviation (SD) or median (inter-quartile range) for continuous variables, and as frequency or percentage for categorical variables. For baseline characteristics analysis, the statistical differences among three groups were tested with t-test or one-way ANOV A for continuous variables and chi-square or fisher test for categorical variables

### Uni- and multivariate logistic analyses of CHD by TyG group

Logistic regression analysis of the CHD risk for tertiles 2 and 3 using tertile 1 as the control group showed that, after the adjustment for age, sex, HTN, DM, and smoking, there was no significant difference in the tertile 2 group (OR, 1.29; 95% CI, 0.77–2.16; P = 0.331), while each 1-unit increase in TyG index was associated with a 2.06-fold increased risk of CHD in the tertile 3 group (OR, 2.06; 95% CI, 1.16–3.65; P = 0.013) (Table 4).

**Table 4:** Univariate and multivariate logistic analyses of CHD in tri-sectional TyG groups

	Non-adjusted		Model I		Model II	
	OR (95%CI)	*P*	OR (95%CI)	*P*	OR (95%CI)	*P*
Tertile 1	Ref.		Ref.		Ref.	
Tertile 2	1.49 (0.93–2.39)	0.095	1.48 (0.91–2.43)	0.116	1.29 (0.77–2.16)	0.331
Tertile 3	3.05 (1.85–5.02)	< 0.001	2.99 (1.77–5.05)	< 0.001	2.06 (1.16–3.65)	0.013

None, non-adjusted model. Model I was adjusted for age and sex. Model II was adjusted for age, sex, hypertension, diabetes mellitus and smoking history

### ROC analyses of TyG index

**Fig. 2:**
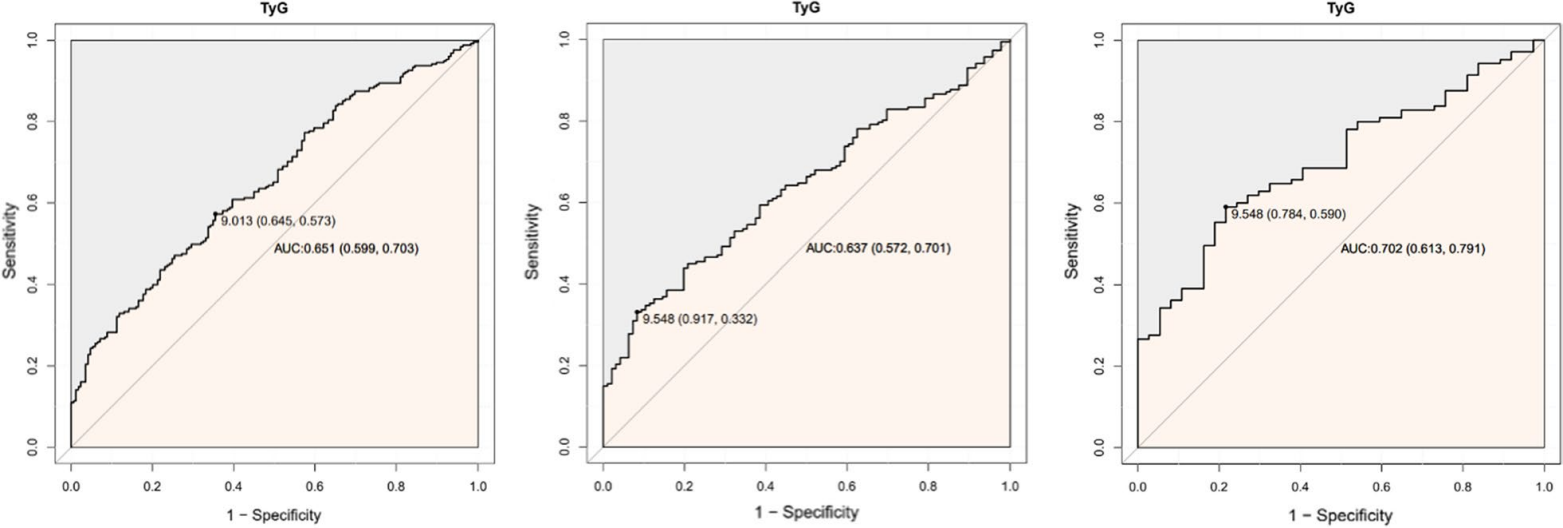
ROC analyses of different levels of TyG index. The rule-in threshold of three ROC was respectively minimum value of TyG (7.91) and the tri-sectional quantiles of TyG (8.78,9.22)

The results of the ROC analysis of the TyG index are summarized in Table 5. The rule-in threshold of TyG index of 9.22 showed the largest AUC (0.702; 95% CI, 0.613–0.791; P < 0.001), and the critical value of the TyG index of CHD was 9.548 (sensitivity, 59.0%; specificity, 78.4%). When the rule-in threshold was 8.78, the AUC was 0.637 (95% CI, 0.572–0.701; P < 0.001), and when the rule-in threshold was 7.91, the AUC was 0.651 (95% CI, 0.599–0.703; P < 0.001) (Fig. 2; Table 5).

**Table 5:** ROC curve analyses of different levels of TyG index

	AUC	95% CI	*P*	Sensitivity	Specificity	Youden’s index
≥ 7.91	0.651	0.599–0.703	< 0.001	0.573	0.645	9.013
≥ 8.78	0.637	0.572–0.701	< 0.001	0.332	0.917	9.548
≥ 9.22	0.702	0.613–0.791	< 0.001	0.590	0.784	9.548

The rule-in threshold of three ROC was respectively minimum value of TyG (7.91) and the tri-sectional quantiles of TyG (8.78, 9.22)

### Association between TyG index and coronary Gensini score

**Fig. 3:**
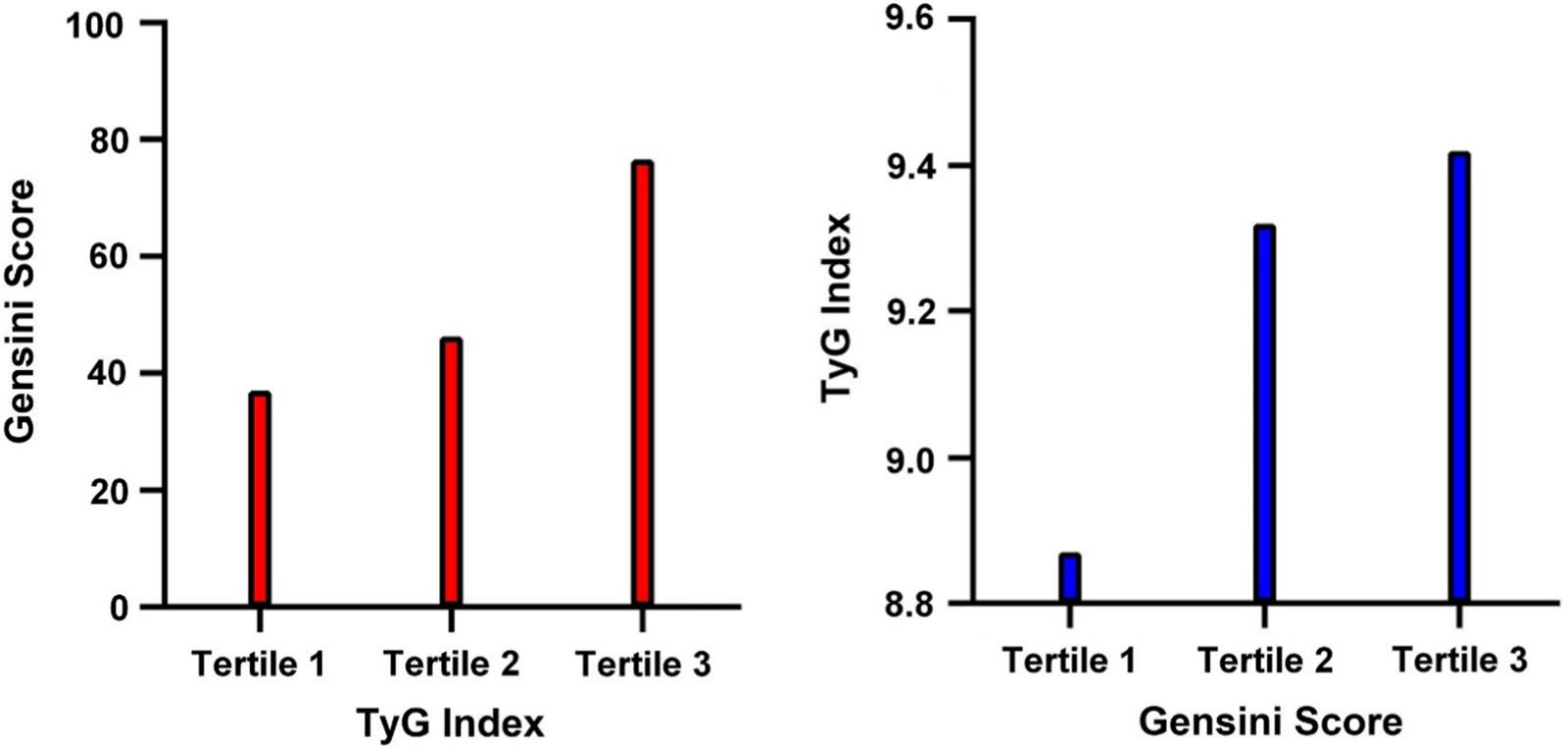
The relationship of Gensini score and TyG in NAFLD-CHD patients. The red bar chart illustrates Gensini score in tri-sectional TyG groups among NAFLD-CHD patients (each group = 85), and the blue bar chart illustrates TyG index in tri-sectional Gensini score groups among NAFLD-CHD patients(each group = 85)

**Fig. 4:**
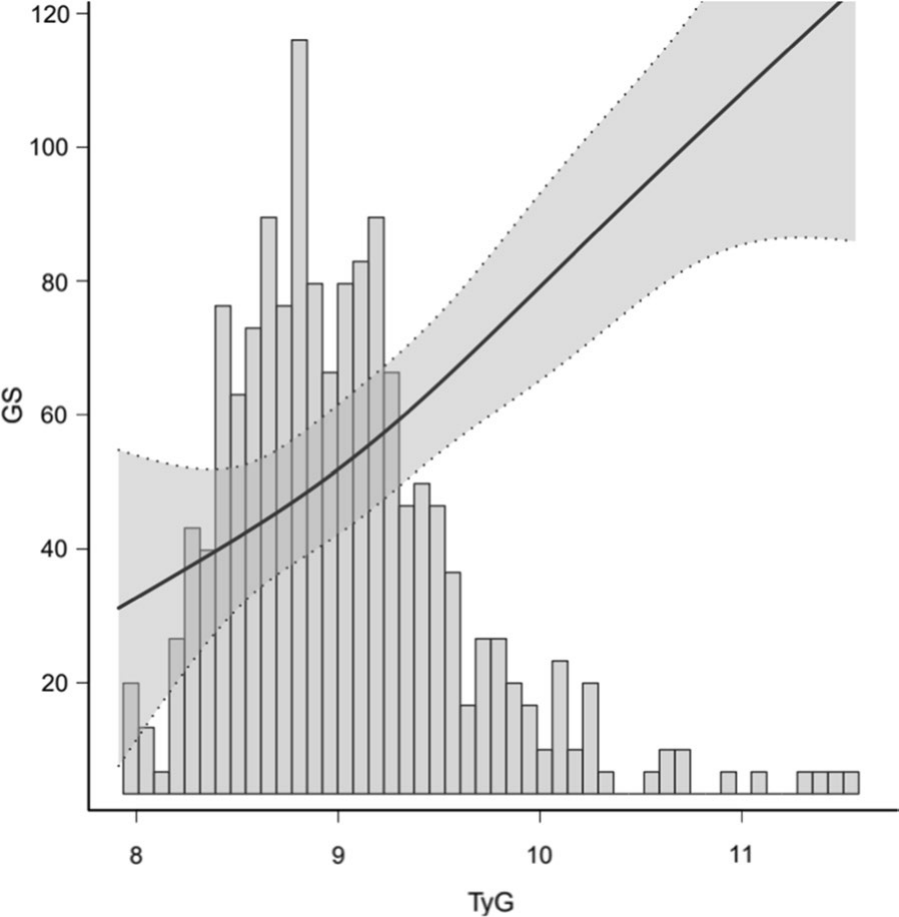
Gaussian graph of the relationship between Gensini score and TyG index

Spearman’s correlation analysis between Gensini score and DBP, AST, TG, HDL-C, FBG, TyG, and TyG-BMI showed that Gensini score was not significantly correlated with DBP (r = 0.143, P = 0.022), AST (r = 0.124, P = 0.048), and HDL-C (r = − 0.170, P = 0.006). Weak relationships were detected between Gensini score and TG (r = 0.305, P < 0.001), FBG (r = 0.253, P < 0.001), and TyG-BMI (r = 0.207, *P* = 0.001). A moderate relationship was demonstrated between Gensini score and the TyG index (r = 0.405, P < 0.001) (Table 5). Figure 3 presents the Gensini score by TyG group among the NAFLD-CHD patients (n = 85 each) and the TyG index by the Gensini score groups among the NAFLD-CHD patients (n = 85 each). Figure 4 shows a Gaussian graph of the positive correlation between Gensini score and TyG index. The multifactor linear regression analysis results are presented in Tables 6, 7. Gensini score was significantly and positively correlated with TyG index. Each 0.1-unit increase in TyG in the NAFLD-CHD group was associated with an increase of 2.44 in Gensini score (β = 2.44; 95% CI, 0.97–3.91; P = 0.002).

**Table 6:** Relationship between Gensini score with DBP, AST, TG, HDL-C, FGB, TyG and TyG-BMI

	*r*	*p*
DBP (mmHg)	0.143	0.022
AST (U/L)	0.124	0.048
TG (mmol/L)	0.305	< 0.001
HDL-C (mmol/L)	− 0.170	0.006
FGB (mmol/L)	0.253	< 0.001
TyG	0.405	< 0.001
TyG-BMI	0.207	0.001

Spearman correlation analysis was used to tset the association of Gensini score and various indicators

**Table 7:** Univariate and multivariate linear regression of Gensini scores

	Non-adjusted			Adjusted		
	*β*	95%CI	*P*	*β*	95%CI	*P*
TyG*0.1	2.24	0.93–3.56	0.001	2.44	0.97–3.91	0.002

None, non-adjusted model. Adjusted model was composed of age, sex, hypertension, diabetes mellitus and smoking history

## Discussion

Increasing studies have demonstrated an association between TyG index and CHD. A recent cohort study of 62,443 Chinese people without CVD showed that changes in the TyG index can predict CVD risk in the general population [20]. In a prospective cohort of 40,473 subjects, Tian et al. [21] reported that different TyG index factors were significantly associated with subsequent CVD risk in normal-weight individuals. Liu et al. [22] followed 96,541 participants in the Kailuan study for a median of 10.33 years and showed that TyG index was an independent risk factor for CVD. In summary, these studies revealed an association between TyG index and CVD, but it is important to note that most of these studies enrolled participants from the general population rather than specific high-risk groups. NAFLD is a common and easily overlooked chronic liver disease with a higher mortality rate than that of the general population, and cardiovascular events are among the most common complications in patients with early NAFLD [23]. A cohort study of 10 761 subjects showed that TyG was able to identify individuals at risk of NAFLD with a higher sensitivity at a threshold of 8.5 [24]. Another cohort study confirmed a positive correlation between TyG index and NAFLD in the general population [25]. In clinical studies, TyG index identified NAFLD and liver fibrosis in overweight and obese people [26, 27]. Given that NAFLD is associated with an increased risk of CVD and that TyG index can predict both NAFLD and CVD, relatively few studies have investigated whether TyG index predicts CHD in NAFLD populations. Therefore, this study aimed to investigate this association.

The precise mechanism of the relationship between TyG index, NAFLD, and CHD has not been fully elucidated, but it is thought to include IR, endothelial dysfunction, inflammation, glucose and lipid metabolism disorders, and thrombosis. The TyG index not only reflects IR, it is closely related to these mechanism [11, 28–30], which seems to explain why a significant number of NAFLD patients and an elevated TyG index ultimately develop CHD.

IR is thought to be a driver of many diseases including NAFLD and CHD. Under physiological conditions, insulin regulates glucose metabolism by processing glucose in insulin-sensitive tissues, while IR involves the decreased sensitivity of tissues to insulin and the impaired regulation of glucose metabolism, leading to the impairment of multiple organ functions, including the liver and heart [31]. Studies have demonstrated that insulin plays a very important role in maintaining vascular contraction and relaxation function stability by exerting nitric oxide–dependent vasodilation and endothelin-1 (ET-1)-dependent vasoconstriction through phosphatidylinositol 3-kinase (PI3K)- and mitogen-activated protein kinase (MAPK)-dependent signaling pathways in the vascular endothelium; physiological concentrations of insulin can maintain the balance between them, while IR is accompanied by damage to the PI3K-NO pathway and enhancement of the MAPK-ET-1 pathway, at which point this balance is disrupted, leading to endothelial dysfunction [32, 33]. As mentioned earlier, endothelial dysfunction is also associated with glucose and lipid toxicity due to abnormal metabolism. These abnormal physiological processes are extremely common in patients with NAFLD and appear to partly explain the high risk of cardiovascular events in patients with NAFLD. Metformin is a common medication that both improves NO-dependent vasodilatation function and reduces ET-1 level; it can also improve IR to some extent. Clinical studies have confirmed that metformin can significantly reduce cardiovascular events in patients with IR, which also supports the important role of IR in NAFLD patients with CHD [34].

Patients with NAFLD exhibit varying degrees of endothelial cell damage. In simple terms, the liver intake of fatty acids and synthesis in patients with NAFLD is affected, glucose production is unchecked, leading to local lipid and glucose production and IR development. IR increases glucose metabolism and lipid metabolism disorders, leading to vascular endothelial cell injury, and previous studies have confirmed that the presence of IR is a major determinant of vascular endothelial disorders after other metabolic factors are considered [35]. At the same time, excessive accumulation of fat in the liver can lead to metabolic stress, which can promote the generation of reactive oxygen species (ROS) in the mitochondria [36]. Excessive ROS in the body can overwhelm the antioxidant system, resulting in the generation of a large number of oxidized low-density lipoproteins, malondialdehyde, and homocysteine in the circulation, leading to endothelial injury and an impaired vascular response [37–39]. Demirci et al. [11] found that TyG index reflected IR and was correlated with endothelial dysfunction. Endothelial cell injury, especially in coronary artery disease, is among the most important initiating factors for coronary atherosclerotic lesion development [40, 41].

NAFLD patients have different degrees of the systemic inflammatory response that is mainly related to macrophage activation and accumulation. Kupffer cells are also involved [42, 43] and closely associated with IR, even in the early stages before insulin secretion becomes impaired [44]. The same mechanism also occurs in obese individuals. An important way that obesity causes low inflammation involves macrophage activation and migration, which can release various inflammatory factors such as interleukin and tumor necrosis factor, creating an inflammatory environment that blocks insulin from doing its job in fat cells and leading to IR, while the Mediterranean diet reportedly improved IR in NAFLD and obese patients, possibly by lowering their inflammation levels [45, 46]. The systemic inflammatory response in patients with NAFLD is closely associated with CHD. On the one hand, it occurs through direct vascular damage induced by inflammatory mediators; on the other hand, IR due to systemic inflammation also increases the risk of CHD [47, 48].

Patients with NAFLD displayed specific changes in the blood lipid profile, mainly characterized by increased TG and decreased HDL-C as well as the excessive circulation of very low-density lipoprotein cholesterol (VLDL-C), which was consistent with our study results. VLDL-C is believed to be the basis of TG and HDL disorders and trigger a series of other plasma lipoprotein abnormalities, such as abnormal numbers and functions of LDL-C, middle-density lipoprotein cholesterol, and apolipoprotein B. Abnormal blood lipid composition usually causes atherosclerotic lesions [49, 50]. It is worth noting that IR plays a huge role in the excessive production of VLDL-C in NAFLD patients. The abnormal expression of key molecules in the insulin signaling pathway, such as phosphatase and protein kinase, leads to hepatic IR, and these signal transduction changes in turn lead to the overexpression of key proteins in lipid metabolism, stimulating the liver to produce large amounts of VLDL-C, which subsequently triggers dyslipidemia [51]. These abnormal fat deposits in the liver or other tissues are often associated with poor metabolism in the heart, increasing the risk of cardiovascular events [52]. In addition, liver diseases directly affect the lipid metabolism. Hepatokines are a class of proteins secreted by the liver that regulate metabolic function and can change the secretion of liver cytokines, thus affecting body metabolism. Several clinical studies have confirmed that some hepatokines, such as fetuin A, leukocyte cell–derived chemotaxin 2, and selenoprotein P, are closely associated with the risk of CHD [53–55].

NAFLD can also lead to thrombosis and embolism. The liver plays a role in maintaining the balance between coagulation and anticoagulation, and NAFLD can directly lead to hypercoagulability, which may be related to imbalance in coagulant and anticoagulant secretion under pathological conditions [56, 57]. Another possible reason for the increased risk of CHD in patients with NAFLD is that IR increases the risk of thrombosis. IR leads to severe endothelial injury, long-term chronic inflammation, coagulation, low fibrinogen levels, and other abnormal states, causing bleeding and coagulation imbalances and promoting thrombosis [58, 59]. Acute platelet thrombosis in the coronary arteries is closely associated with acute myocardial infarction and often leads to a poor prognosis, which seems to explain the primary cause of early death in NAFLD patients with cardiovascular events.

In summary, IR plays an important role in the occurrence of cardiovascular events in NAFLD patients; at the same time, endothelial cell damage, inflammation, disorders of glucose metabolism and lipid metabolism, thrombosis, and embolism also facilitate the process. The TyG index is related to the levels of triglyceride and glucose in the body, pathological processes that can lead to increased triglyceride and glucose levels, thus promoting the increase of TyG level, which can explain that the TyG index is related to the occurrence of CHD in NAFLD patients and reflects disease severity. The disorder of glucose and lipid metabolism in patients with NAFLD promotes the occurrence of IR, and IR-induced vascular endothelial injury becomes the core link of CHD in patients with NAFLD.

Accordingly, inflammation and oxidative stress, glucose metabolic abnormalities, and endothelial injury create favorable conditions for abnormal blood lipid deposition, thus promoting the occurrence of atherosclerotic lesions. Progressive vascular stenosis eventually leads to chronic CHD, bleeding, and clotting imbalances, increasing the risk of thrombosis and plaque rupture and often inducing acute myocardial infarction. The correlation of NAFLD and a high CHD risk is an objective phenomenon that cannot be completely explained simply by several mechanisms. It is often caused by endothelial injury, IR, inflammation, and abnormal glucose and lipid metabolism. These phenomena promote each other, often forming a vicious circle that eventually leads to disease progression in which endothelial injury is recognized as the core link and IR is a key factor in mediating endothelial injury.

This study found that higher TyG index levels were associated with an increased risk of CHD in NAFLD populations despite the adjustment for traditional CHD risk factors such as age, sex, smoking, HTN, and DM. We also found that the TyG index was positively correlated with coronary artery disease severity in patients with NAFLD and CHD. In this study, TyG, a new IR indicator, was used to emphasize the important role of IR in the occurrence of CHD in NAFLD patients, providing a new way to indicate CHD and coronary artery disease severity among NAFLD patients.

This study also has its limitations. First, it was a cross-sectional study of small samples; the single-center and small sample size may cause bias; at the same time, the research and control groups were not strictly matched; the multivariable logistic regression analysis adjusted for common confounding factors but did not completely offset the differences between groups. Second, patients with severe NAFLD, such as those with liver fibrosis and cell necrosis, were not included in this study, which may have led to an underestimation of the efficacy of the TyG index in predicting CHD. Third, we were unable to assess the risk of impaired glucose homeostasis for CHD in NAFLD patients due to the lack of data on baseline glycosylated hemoglobin and 2-h oral glucose tolerance test results in a significant number of patients. Finally, since this was a cross-sectional study, its findings can indicate that the TyG index is positively correlated with the occurrence of CHD in NAFLD patients but cannot claim to have predictive value. Future large-scale multi-center prospective studies are required to further verify the predictive power of the TyG index on CHD risk among NAFLD patients.

## Conclusions

Our study found that the TyG index of patients with NAFLD combined with CHD was significantly higher than that of patients with NAFLD alone, and the TyG index was positively correlated with coronary artery disease severity. The TyG index is an emerging index reflecting IR that differs from previous evaluation standards. Because of its simple, cheap, and reliable characteristics, it can be widely used in primary hospitals and communities. It can be used as a supplement to the classic risk factors for CVD, especially in high-risk cardiovascular populations, such as those with NAFLD, and the correlation may be higher and reflect lesion severity to some extent, but this requires further confirmation in larger studies.

## Data Availability

The original contributions of this study are included in the article/supplementary material. Further inquiries can be directed to the corresponding author.

## Abbreviations

ALT: Alanine aminotransferase
AST: Aspartate aminotransferase
CHD: Coronary heart disease
CVD: Cardiovascular disease
DBP: Diastolic blood pressure
DM: Diabetes mellitus
FBG: Fasting blood glucose
HDL-C: High-density lipoprotein cholesterol
HTN: Hypertension
IR: Insulin resistance
LDL-C: Low-density lipoprotein cholesterol
NAFLD: Non-alcoholic fatty liver disease
Scr: Serum creatinine
TC: Total cholesterol
TG: Triglyceride
TyG: Triglyceride-glucose
